# N^ε^-acetyl-*β*-lysine or glycine betaine as compatible solutes in response to increasing ammonia in *Methanoculleus sp* strains

**DOI:** 10.1093/femsle/fnaf143

**Published:** 2025-12-24

**Authors:** Anna Schnürer, Maria Westerholm, Anders Broberg

**Affiliations:** Department of Molecular Sciences, Swedish University of Agricultural Sciences, Uppsala BioCenter, Box 7025, SE-750 07 Uppsala, Sweden; Department of Molecular Sciences, Swedish University of Agricultural Sciences, Uppsala BioCenter, Box 7025, SE-750 07 Uppsala, Sweden; Department of Molecular Sciences, Swedish University of Agricultural Sciences, Uppsala BioCenter, Box 7025, SE-750 07 Uppsala, Sweden

**Keywords:** methanogen, ammonia stress, compatible solute, glycine betaine, N^ε^-acetyl-β-lysine, HR-MAS NMR

## Abstract

Methanogens rely on compatible solutes to withstand osmotic stress, yet their responses to high ammonium concentrations, common in biogas digesters, remain poorly understood. In this study, intracellular osmolyte accumulation was examined in four *Methanoculleus bourgensis* strains (MAB1, MAB2, MAB3, and BA1), isolated from high-ammonia biogas digesters, under progressive increase in concentrations of ammonium and sodium chloride. Their responses were compared with those of the type strain *Methanoculleus bourgensis* MS2^T^ and the halophilic *Methanoculleus submarinus* Nankai-1^T^. All investigated strain grew to 12 g l^−1^ NH_4_^+^-N (0.3 mg l^−1^ NH_3_), and gradual adaptation increased ammonium/ammonia tolerance in some strains to 25 g l^−1^ NH_4_^+^-N. Whereas the reference strains accumulated glycine betaine under both ammonium and sodium chloride stress, the *M. bourgensis* strains from high ammonia biogas systems uniquely accumulated N^ε^-acetyl-β-lysine during increasing levels of ammonium chloride. This β-amino acid derivative is known as a NaCl-induced osmoprotectant in methanogens, but it´s association with high ammonium/ammonia levels in pure cultures has not previously been demonstrated. Our findings identify N^ε^-acetyl-β-lysine biosynthesis as a potential mechanism underpinning the exceptional ammonium/ammonia tolerance of *M. bourgensis*, a taxon frequently dominating methane production in high-ammonia biogas systems, while also revealing notable variation in this trait among its subspecies.

## Teaser

The osmolyte NÎµ-acetyl-Î²-lysine is produced by Methanoculleus sp in repsonse to high ammonia levels.

## Introduction

Anaerobic digestion (AD) is an established biological process used to treat a wide range of organic waste materials while simultaneously generating renewable energy in the form of biogas and producing a nutrient-rich digestate suitable for use as fertilizer (Kougias et al. 2018). The degradation of organic matter during the AD proceed via four main interdependent steps, hydrolysis, acidogenesis, acetogenesis, and methanogenesis, performed by different groups of microorganisms (Schnürer 2016).

When protein-rich substrates, such as food waste and animal manure are degraded, ammonium is released. Although, ammonium serves an essential nutrient for many microorganisms (Merrick et al. 1995) high concentrations can inhibit key populations. Elevated ammonium levels particularly inhibit acetate-utilizing (aceticlastic) methanogens, resulting in volatile acid accumulation and reduced methane production (Schnürer et al. 2008, Duan et al. 2022, Wang et al. 2022). In aqueous solution, ammonium exists in two forms, unionized free ammonia (NH_3_) and the ammonium ion (NH_4_^+^). NH_3_ is generally considered more toxic as it freely diffuses across cell membranes. The proportion of NH_3_ vs NH_4_^+^ is increased with higher temperature and pH (Capson-Tojo et al. 2020). Once, inside the cells, NH_3_ is converted to NH_4_^+^, disrupting intracellular pH homeostasis and increasing osmotic pressure, which can lead to excessive water influx, leading to cell swelling and, potentially, lysis (Sprott et al. 1986, Bremer et al. 2019,).

All microorganisms must maintain an intracellular osmotic pressure slightly higher than that of their surrounding environment. Under high-salinity conditions, they deploy several adaptive strategies (Welsh 2000). One strategy is the “salt-in” strategy, which involves the accumulation of inorganic ions, such as potassium (e.g K^+^), to balance osmotic pressure. Another widely used strategy is synthesis or by uptake of organic compatible solutes (osmolytes), thereby stabilising cellular functions without interfering with core metabolic processes (Lai et al. 1991, Roesser et al. 2001, Salma et al. 2020). Common osmolytes, collectively termed compatible solutes, include polyols (e.g. glycerol and mannitol), low molecular-weight nonionic carbohydrates (e.g. sucrose, trehalose, and glucose), amino acids and their derivatives (e.g. proline, glutamate, and glycine), organic zwitterions (e.g. ectoine), methylamines (glycine betaine). The compounds are remained in high intracellular concentrations to balance the osmotic pressure and to protect intracellular enzymes and organelles from stress induced damage (Welsh 2000). For methanogens, uptake of potassium, iron and molybdenum and upregulation of genes responsible for nitrogen assimilation and anti-oxidative stress has been proposed as potential mitigation strategies (Sprott and Patel 1986, Kato et al. 2014, Wang et al. 2015, Duan et al. 2022, Feng et al. 2022). Production of compatible solutes in response to high sodium levels is well known for methanogens, of which some are predominantly (or even uniquely) used by Archaea. One example is the production of β-amino acid derivatives (e.g. N^ε^-acetyl-β-lysine and β-glutamine), having the benefit of not interacting with the metabolic or biosynthetic machinery in the cells (Martin et al. 1999). However, information on compatible solute production by ammonia-tolerant methanogens in response to elevated ammonium/ammonia concentrations remains scarce. A deeper understanding of the adaptive strategies employed by hydrogenotrophic methanogens is essential for elucidating mechanisms underlying resilience under high-ammonia conditions.

*Methanoculleus bourgensis* is a hydrogenotrophic methanogen that reduces CO_2_ to methane (CH_4_) using H_2_ or formate as electron donors. This species is prevalent in AD processes exposed to elevated ammonium/ammonia levels (Maus et al. 2015, Halim et al. 2021, Lee et al. 2021). It is also frequently identified as a suitable syntrophic partner for acetate oxidation (Westerholm et al. 2016), underscoring its pivotal role in sustaining efficient biogas production under ammonia stress. Moreover, bioaugmentation with *Methanoculleus* sp. enrichments has demonstrated the ability to counteract ammonia inhibition in biogas processes (Fotidis et al. 2017, Tian et al. 2019). Despite their frequent occurrence in ammonia-stressed biogas digesters, neither the extent of their ammonia tolerance nor the mechanisms enabling them to endure ammonia stress has been elucidated.

The aim of this study was therefore to identify and quantify intracellular accumulation of major organic osmolytes in four strains of *M. bourgensis*, isolated from high-ammonia biogas digesters, and to compare their response with that of the type strain *M. bourgensis* MS2^T^, originating from a biogas digester operating under low-ammonia conditions. In addition, the halophilic *Methanoculleus submarinus* Nankai-1^T^ was included as a sodium-salt-tolerant representative of the genus. To link osmolyte profiles to ammonia tolerance, all methanogens were cultivated across a range of ammonium/ammonia levels, and the effects of successive adaptation were assessed.

## Materials and methods

### Methanogenic strains and cultivation

The methanogenic strains included in the present study were *M. bourgensis* strain MAB1, MAB2, MAB3, and BA1, all of which were isolated in-house from four different anaerobic digesters operating at high ammonia concentrations (∼6 g l^−1^ NH_4_^+^-N) (Schnürer et al. 1999). These strains were selected based on the apparent ammonia/ammonium tolerance. The type strains of *M. bourgensis* MS2^T^ (DSM 3045) and *M. submarinus* Nankai-1^T^ (DSM 15122) were ordered from the German Collection of Microorganisms and Cell Cultures (DSMZ, Leibniz, Germany). *M. bourgensis* MS2^T^ was originally isolated from anaerobic digester degrading tannery by-products (initially inoculated with digested sewage sludge) (Ollivier et al. 1986). The halophilic *M. submarinus* Nankai-1^T^ was isolated from saline deep marine sediments (Mikucki et al. 2003). All strains were cultivated in reduced basal medium complemented with 0.2 g l^−1^ yeast extract and 5 mM sodium acetate and prepared as described previously by (Westerholm et al. 2010). Cultivation was performed either in 20 ml or 200 ml aliquots in 118 ml or 1 l serum bottles, respectively. After inoculation of the methanogen, 0.8 atm pressure of H_2_/CO_2_ (80/20 v/v) was added as main carbon and energy source. The cultures were incubated in the dark, at 37°C and without shaking (shaking resulted in less efficient growth).

### Growth experiments to resolve ammonia tolerance

The ability of the strains to adapt to ammonium/ammonia was assessed for all investigated strains through a gradual increase of ammonium chloride concentration at each transfer. For each successive step, the growth medium contained 0.3, 11.4, 22.8, 34.2, 45.6, 57, 68.4, 79.8, and 91.2 g l^−1^ NH_4_Cl to reach ammonium-nitrogen concentrations of 0.1, 3, 6, 9, 12, 15, 18, 21 and 25 g l^−1^ NH_4_^+^-N. For MAB1, MAB2, MAB3, and BA1, analysis of ammonia tolerance without gradual adaptation was also studied by directly transfer of inoculum from medium containing 0.3 g l^−1^ NH_4_Cl to the different ammonia levels. The pH of the medium was 7.2–7.3, independent of ammonium concentration resulting in ammonia levels between 25–299 mg l^−1^. Methane production was analysed throughout the experimental trials using a Clarus 500 gas chromatograph (GC) equipped with a 7’ HayeSep N 60/80, 1/8” SF column and a FID detector. Helium was used as carrier gas, at a flow rate of 31 ml/min. The column and the detector were operated at 60 and 250°C, respectively and, the injection was carried out at 40°C. The obtained growth curves were linear and thus the growth rate was evaluated by calculating the increase in methane concentration over time.

### Identification of compatible solutes

High-resolution magic-angle spinning nuclear magnetic resonance (HR-MAS NMR) was used to analyse intact cells of the strain *M. bourgensis* BA1. Culture suspensions (500 ml, OD_600 nm_ ∼0.2), grown with 0.1 and 12 g l^−1^ NH_4_^+^-N, were centrifuged at 4°C (12 500 × g) and aliquots of the pellets were suspended in 20 µl D_2_O. The suspensions were kept on ice for about 1 h to allow precipitated iron sulfide to settle as described before (Houwen et al. 1987). Aliquots of the suspension were transferred to 4-mm high-resolution magic-angle spinning nuclear magnetic resonance (HR-MAS NMR) rotors (Zirconia, Bruker, Germany). Samples were analyzed by HR-MAS NMR at 600 MHz (Bruker DRX-600; Bruker SpectroSpin, Germany) using a HR-MAS SB BL4 probe head, at the spinning rate 5000 Hz. One-dimensional T_2_-filtered ^1^H-NMR data was acquired using a Carr–Purcell–Meiboom–Gill pulse sequence [90°−(τ−180°−τ)_n_ -acquisition, τ=300 µs, n = 100; (Meiboom et al. 1958)] with 0.5 s relaxation delay. Standard manufacturer supplied pulse sequences were used for two-dimensional (COSY and TOCSY) HR-MAS ^1^H-NMR experiments.

A cell pellet from a 200-ml culture of *M. bourgensis* BA1 grown in 12 g l^−1^ NH_4_^+^-N, was extracted with 80% aqueous CH_3_OH (400 ml) in an ultrasonic bath (30 min). Following centrifugation, the supernatant was dried in a vacuum centrifuge and the dried residue dissolved in D_2_O/CD_3_OD (3:2, 0.7 ml) and analyzed by NMR at 600 MHz (Bruker DRX-600; 5-mm QXI probe head; Bruker SpectroSpin, Germany), using manufacturer supplied pulse sequences for one-dimensional ^1^H-NMR experiments, and two-dimensional (COSY, TOCSY, HSQC, HMBC) ^1^H-^1^H and ^1^H-^13^C NMR experiments.

Cultures of all investigated methanogens, strain BA1, MAB1, MAB2, MAB3, MAB5, *M. submarinus*, and *M. bourgensis* MS2^T^, were cultivated with or without additions of 12 g l^−1^ NH_4_^+^-N or Na^+^ and analyzed by LC-MS. Culture samples were pelleted in 1.5 ml plastic tubes, and the pellets were extracted with CH_3_OH for 1 h in an ultrasonic bath. After centrifugation, samples (5 µl) of the supernatants were injected on a 100 × 2.1 mm HyperCarb porous graphitic column (3 µm; ThermoQuest Runcorn, Cheshire, United Kingdom), eluted with a gradient of CH_3_OH in H_2_O (0% during 3 min, and then a linear gradient to 40% CH_3_OH in 5 min, followed by 8 min at 40% CH_3_OH, in the presence of 0.1% TFA) at 0.2 ml min^−1^. The column was connected to an electrospray ionization mass spectrometer (Bruker Esquire-LC; Bruker Daltonics, Germany) operated in the positive ion mode.

### Cultures for quantification of glycine betaine and N^ε^-acetyl-β-lysine

*M. bourgensis* MAB2 was used for a quantitative analysis of accumulation of intracellular organic compounds during growth in various ammonium and with sodium salt. The methanogen was cultured in medium containing 0.1, 3, 6, and 12 g l^−1^ NH_4_^+^-N or 12 g l^−1^ Na^+^ (30.4 g l^−1^ NaCl). The methanogens *M. bourgensis* MS2^T^, closely related to *M. bourgensis* MAB2 and the halophilic *M. submarinus* Nankai-1^T^ were included for comparison and these strains were analysed after growth in medium including 0.1 or 12 g l^−1^ NH_4_^+^-N or 12 g l^−1^ Na^+^. All analysed cultures had grown at the selected conditions for at least one transfer, and all treatments were done in triplicate.

### Quantification of glycine betaine and N^ε^-acetyl-β-lysine

Culture suspensions of *M. bourgensis* sp. MAB2, *M. bourgensis* sp. MS2^T^, and *M. submarinus* Nankai-1^T^ were centrifuged in two steps in 1.5 ml plastic tubes (1 + 1 ml) and the supernatants were discarded. The pellets were stored under CH_3_OH (100 µl) at -80°C until analyzed. Subsequently, the pellets were extracted with CH_3_OH (1000 µl), containing 0.19 µg and 3.24 µg, respectively, of the deuterium labelled internal standards (glycine betaine) and (N^ε^-acetyl-β-lysine), in an ultrasonic bath for 1 h, in the original 1.5-ml plastic tubes. Following centrifugation, the supernatants were dried in a vacuum centrifuge. The dried residues were dissolved in 200 µl H_2_O and were subjected to LC-MS analysis. Samples (10 µl) were injected on a 100 × 4.2 mm HyperCarb porous graphitic column (3 µm; ThermoQuest Runcorn, Cheshire, United Kingdom), eluted with a gradient of CH_3_OH in H_2_O (0% during 3 min, and then a linear gradient to 40% CH_3_OH in 5 min, followed by 8 min at 40% CH_3_OH, in the presence of 0.1% TFA) at 0.7 ml min^−1^. The column was connected to a UV-detector (210 nm) and to an electrospray ionization mass spectrometer (Bruker Esquire-LC; Bruker Daltonics, Germany) operated in the positive ion mode. Ion chromatograms were constructed from ions at *m/z* 118.1 ± 0.3 (glycine betaine), 127.1 ± 0.3 (glycine betaine**-***d_9_*), 189.1 ± 0.3 (N^ε^-acetyl-β-lysine), and 192.1 ± 0.3 (N^ε^-acetyl-*d_3_*-β-lysine), and peaks at 2.3 min (glycine betaine and glycine betaine**-***d_9_*) and 10.8 min (N^ε^-acetyl-β-lysine and N^ε^-acetyl-*d_3_*-β-lysine) were integrated. By direct comparison of the peak integrals for glycine betaine and N^ε^-acetyl-β-lysine, with their respective deuterated reference compound, and by knowledge of the added amounts of the deuterated reference compounds, the glycine betaine and N^ε^-acetyl-β-lysine concentrations were calculated. Finally, the average concentrations of the compounds for the different treatments were calculated and expressed as µg ml^−1^ original culture. Statistical analysis was done in MiniTab 22.3 (MiniTab, LLC, State College, PA, USA). Peaks in ion chromatograms with signal-to-noise ratio < 10 were considered too small for quantification.

### Absolute configuration of N^ε^-acetyl-β-lysine

A small portion of the dry CH_3_OH extract above was treated with 6 M HCl (aq) for 20 h at 110°C, and subsequently dried in a vacuum centrifuge. Organic acids in the sample were then esterified by the addition of 200 µl (*2S*)-2-butanol/AcCl (10:1, 100°C, 1 h). Following drying under a stream of N_2_, the sample was treated with 200 µl perfluoropropanoic anhydride (100°C, 1 h). After drying (N_2_), the sample was dissolved in EtOAc and analyzed by GC-MS [HP5890/5970, HP-5MS (30 m × 0.25 mm, 0.25 µm), 150°C for 5 min, 150–170°C at 5° min^−1^, 170°C for 5 min, carrier gas He at 1 ml min^−1^. The GC-MS data was compared to data obtained on *N^ε^-Cbz-N^β^-tBoc-L-β-lysine* treated similarly (including acidic hydrolysis) but esterified with (*2S*)-2-butanol or 2-butanol. [Sample (*2S*)-2-butyl derivative: 20.30 min; Standard (*2S*)-2-butyl derivative and (*2R*)-2-butyl derivative: 20.33 min; EI-MS (sample and standards): *m*/*z* 438 [M—C_4_H_8_]^+^ (11%), 421 [M—C_4_H_9_O·]^+^ (29%), 258 (31%), 234 (49%), 230 (32%), 229 (39%), 216 (100%), 57 (39%)].

## Results

### Ammonia tolerance of different *Methanoculleus* strains and species

**Figure 1.**
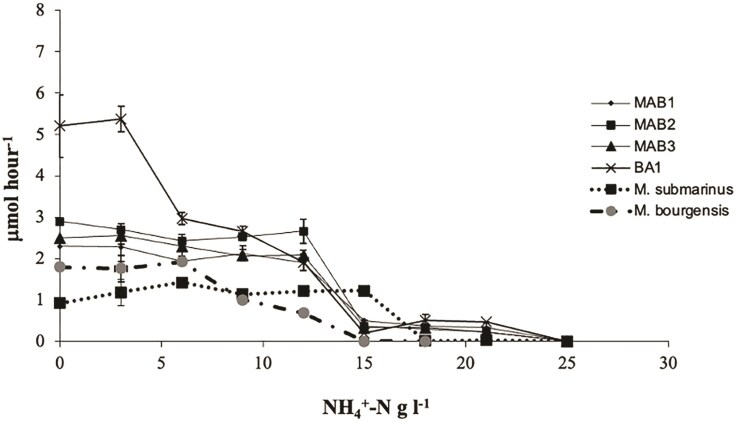
Methane production by six strains of *Methanoculleus* sp.; *Methanoculleus bourgensis* strain MAB1, MAB2, MAB3, and BA1 originating from high ammonia biogas processes, and the type strains *Methanoculleus bourgensis* MS2^T^ and *Methanoculleus submarinus* Nankai-1^T^, at different concentrations of NH_4_^+^-N (0.1-25 g l^−1^).

Evaluation of ammonia tolerance of the difference *Methanoculleus* strains (MAB1, MAB2, MAB3, and BA1), all isolated from high-ammonia biogas systems, showed a reduced methane production rate at ammonia levels exceeding 8–10 g l^−1^ NH_4_^+^-N, and methane production ceased above 12 g l^−1^ NH_4_^+^-N. However, when the ammonia concentration was gradually increased at each transfer, the strains exhibited increased tolerance, maintaining their initial methane production rate at ammonia levels of up to 12 g l^−1^ NH_4_^+^-N (Fig. 1). Below 6 g l^−1^ NH_4_^+^-N, strain BA1 displayed a high methane production rate as compared to the other investigated strains, but between 9–12 g l^−1^ NH_4_^+^-N the rate was similar for all strains. All four strains ceased their methane production at 25 g l^−1^ NH_4_^+^-N. For the reference strain *M. bourgensis* MS2^T^, which was also subjected to successive adaptation, decreased methane production rates were observed at ammonium level exceeded 6 g l^−1^ NH_4_^+^-N, and methane production ceased above 12 g l^−1^ NH_4_^+^-N (Fig. 1). In contrast, the halophilic *M. submarinus* Nankai-1^T^ maintained a stable methane production rate across 0.1-15 g l^−1^ NH_4_^+^-N, whereas no methane was produced at 18 g l^−1^ NH_4_^+^-N or higher (Fig. 1).

### Identification of compatible solutes

**Figure 2.**
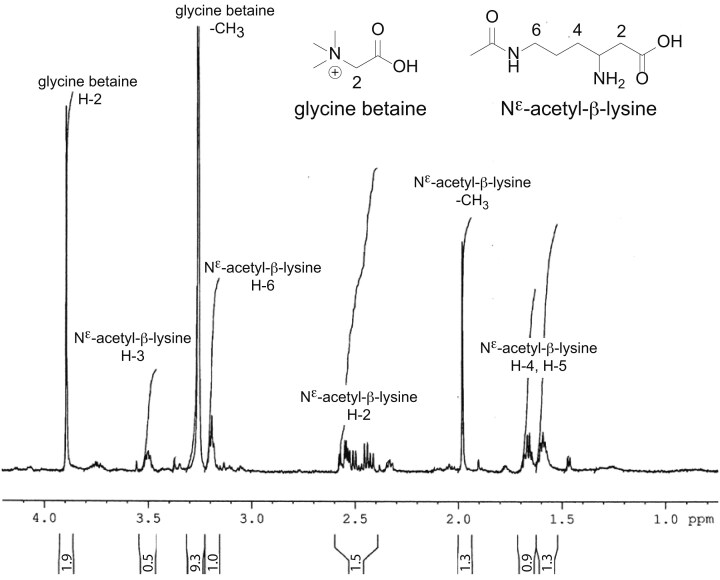
One-dimensional T_2_-filtered high-resolution magic-angle-spinning ^1^H-NMR spectrum of intact cells of *Methanoculleus bourgensis* BA1. The spectrum was recorded at 600 MHz at spinning rate 5000 Hz, using a Carr-Purcell-Meiboom-Gill pulse sequence (Meiboo et al. 1958). The signals from the two main components, glycine betaine and N^ε^-acetyl-β-lysine, are indicated, along with relative signal areas.

An initial analysis of compatible solutes produced in response to high ammonium levels were made with HR-MAS NMR analysis of intact cells of *M. bourgensis* BA1, grown at 12 g l^−1^ NH_4_^+^-N. This analysis resulted in ^1^H-NMR spectra dominated by signals from two main low-molecular-mass metabolites (Fig. 2). One compound gave two prominent ^1^H-NMR singlet signals at δ 3.29 and δ 3.88, with the approximate relative ratio 9:2 (Fig. 2). NMR analysis of a CH_3_OH extract of a comparable culture verified the presence of these signals. The hydrogens giving the singlet signals at δ 3.29 and δ 3.88 from HR-MAS NMR (Fig. 2), were shown by HSQC experiments to be linked to carbon atoms with the chemical shifts δ 54.0 (CH_3_) and δ 67.0 (CH_2_), respectively. HMBC experiments showed a cross-peak from the CH_2_ group to the methyl carbon at δ 54.0 as well as to a carbonyl group at δ 169.1, possibly a carboxylic acid group. The ratio 9:2 for the ^1^H-NMR signals (i.e. 1 × -C*H*_2_- and 3 × -C*H*_3_), along with the presence of carbonyl group in the compound, strongly suggested the compound to be glycine betaine, which was supported by literature NMR data (Weinisch et al. 2018).

The second compound was shown by a HR-MAS NMR COSY experiment to contain the structural motif -CH_2_-CH-CH_2_-CH_2_-CH_2_- (δ 2.44/2.58, δ 3.48, δ 1.69, δ 1.61, and δ 3.20, respectively (Fig. S1). There was also a signal from an acetyl group, possibly in the same molecule (-C*H*_3_, δ 1.98), as judged by the relative signal integrals (Fig. 2). HSQC experiments on CH_3_OH extracts detected the ^13^C NMR shifts for the structural motif -CH_2_-CH-CH_2_-CH_2_-CH_2_- to be δ 38.8, δ 50.0, δ 30.6, δ 25.6, and δ 39.7, respectively, and δ 22.7, for the acetyl methyl group. Moreover, HMBC experiments confirmed the methyl group to be connected to a carbonyl at δ 174.1, and that this carbonyl was at a three-bond distance from the protons of the -CH_2_- group at δ 3.20, as well as that a carboxylic acid group (δ 177.6) was linked to the

-CH_2_- group at δ 2.44/2.58. Finally, the ^13^C- and ^1^H-NMR chemical shifts of the compound was in accordance with the presence of two nitrogen atoms; one amino nitrogen linked to the

-CH- group and one amide nitrogen linked to the -CH_2_- at δ 3.20. These findings suggested the presence of N^ε^-acetyl-β-lysine, which was supported by literature NMR-data (Sowers et al. 1990, Triadó-Margarit et al. 2011, Weinisch et al. 2018). To establish if N^ε^-acetyl-β-lysine was present in L- or D-form, diastereomeric esters with R/S-2-butanol were prepared and analyzed by GC-MS, but this failed to give separation between the two isomers.

**Figure 3.**
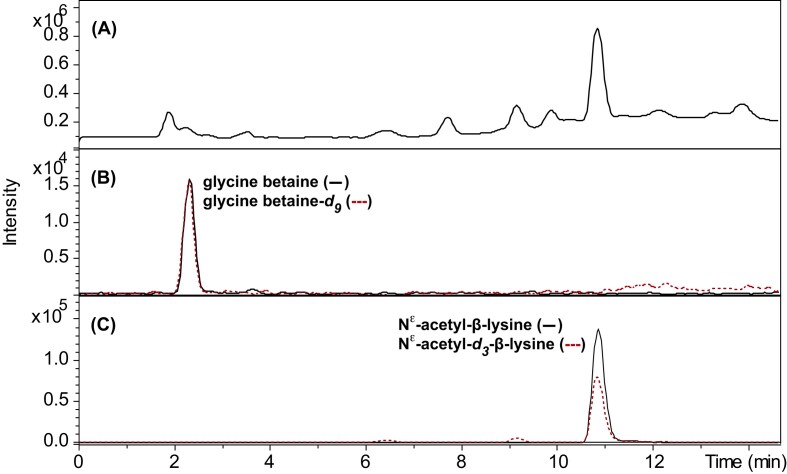
LC-MS analysis of a *Methanoculleus bourgensis* MAB2 culture extract, for identification and quantification of glycine betaine and N^ε^-acetyl-β-lysine. Pelleted culture suspensions were extracted with CH_3_OH containing the deuterium labelled standards glycine betaine-*d*_9_ and N^ε^-acetyl-*d*_3_-β-lysine, respectively, in an ultrasonic bath. Samples were centrifuged and then analyzed on a HyperCarb porous graphitic column, eluted with a gradient of methanol in water in the presence of 0.1% trifluoroacetic acid. The column was hyphenated with an ion-trap MS via an electrospray ion source, and positive mode MS-data was collected. (A) Total ion chromatogram. (B) Extracted-ion chromatograms for glycine betaine (*m/z* 118.1 ± 0.3) and glycine betaine-*d_9_*(*m/z* 127.1 ± 0.3). (C) Extracted-ion chromatograms for N^ε^-acetyl-β-lysine (*m/z* 189.1 ± 0.3) and N^ε^-acetyl-*d_3_*-β-lysine (*m/z* 192.1 ± 0.3).

**Figure 4.**
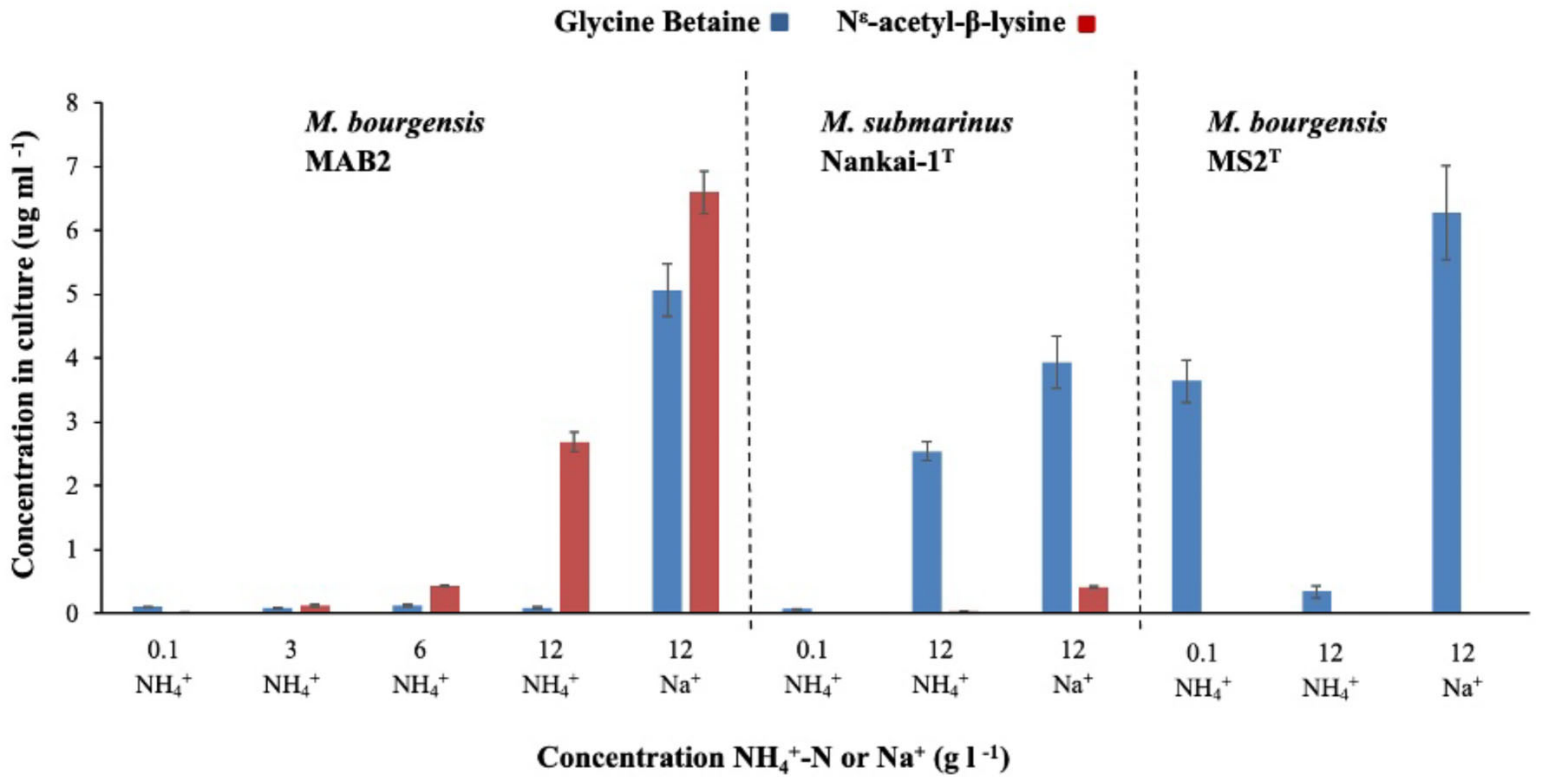
Production of glycine betaine and N^ε^-acetyl-β-lysine by *Methanoculleus bourgensis* MAB2, *M. submarinus* Nankai-1^T^, and *M. bourgensis* MS2^T^, in media with different levels of ammonium or sodium salts. The concentrations were determined by LC-MS using the deuterium labelled internal standards glycine betaine-*d_9_* and N^ε^-acetyl-*d*_3_-β-lysine. Error bars are standard deviations. In short, pelleted culture suspensions were extracted with CH_3_OH containing known concentrations of the deuterium labelled standards glycine betaine-*d*_9_ and N^ε^-acetyl-*d*_3_-β-lysine, respectively, in an ultrasonic bath. Samples were centrifuged and then analyzed on a HyperCarb porous graphitic column, eluted with a gradient of methanol in water in the presence of 0.1% trifluoroacetic acid. The column was hyphenated with an ion-trap MS via an electrospray ion source, and positive mode MS-data was collected. Extracted-ion chromatograms were created for glycine betaine and N^ε^-acetyl-β-lysine, and the respective deuterated standard compounds. Comparison of peak areas for natural and deuterated compounds gave the concentrations for glycine betaine and N^ε^-acetyl-β-lysine.

**Figure 5.**
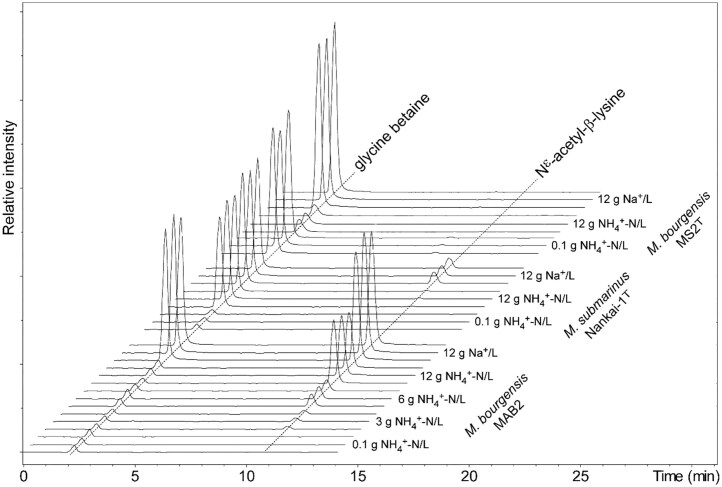
Combined extracted-ion chromatograms for glycine betaine (*m/z* 118.1 ± 0.3) and N^ε^-acetyl-β-lysine (*m/z* 189 ± 0.3) from LC-MS analysis of three strains of *Methanoculleus* sp. cultivated in different concentrations of ammonium and sodium salts. Pelleted culture suspensions were extracted with CH_3_OH containing the deuterium labelled standards glycine betaine-*d*_9_ and N^ε^-acetyl-*d*_3_-β-lysine, respectively, in an ultrasonic bath. Samples were centrifuged and then analyzed on a HyperCarb porous graphitic column, eluted with a gradient of CH_3_OH in water in the presence of 0.1% trifluoroacetic acid. The column was hyphenated with an ion-trap MS via an electrospray ion source, and positive mode MS-data was collected, and extracted-ion chromatograms were constructed for the compounds.

The NMR based identification of glycine betaine and N^ε^-acetyl-β-lysine in BA1, was corroborated by LC-MS experiments on a CH_3_OH extract, which showed chromatographic peaks (2.4 min and 15.0 min, respectively) with major ions at *m/z* 118.1 [M^+^] for glycine betaine, and *m*/*z* 189.1, [M + H]^+^ for N^ε^-acetyl-β-lysine. Furthermore, additional analysis of CH_3_OH extracts of all methanogens, i.e. BA1, MB, MS, MAB1, MAB2, MAB3, and MAB5, detected glycine betaine and N^ε^-acetyl-β-lysine in all cultures, except for *M. bourgensis* MS2^T^, where only glycine-betaine could be detected (Table 1, Figs 3–5, Fig S2).

**Table 1.** Concentrations of glycine betaine and N^ε^-acetyl-β-lysine in cultures of three *Methanoculleus* sp. strains, as determined by LC-MS using deuterium labelled internal standards for quantification.

Organism	NH_4_^+^-N (g ^−1^)	Na^+^ (g ^−1^)	glycine betaine (µg ml^−1^)		N^ε^-acetyl-β-lysine (µg ml^−1^)	
*M. bourgensis* MAB2	0.1	-	0.10	±0.008^a^	0.010	±0.002^b^
	3	-	0.085	±0.005	0.12	±0.013*
	6	-	0.12	±0.011	0.43	±0.018*
	12	-	0.091	±0.014	2.7	±0.16*
	-	12	5.1	±0.41**	6.6	±0.33^b^,**
*M. submarinus* Nankai-1^T^	0.1	-	0.077	±0.003^c^	n.d.^d^	
	12	-	2.5	±0.15*	0.038	±0.001^b^,*
	-	12	3.9	±0.42	0.42	±0.016**
*M. bourgensis* MS2^T^	0.1	-	3.6	±0.33	n.d.^b^	
	12	-	0.34	±0.098^c^,*	n.d.	
	-	12	6.3	±0.74^c^,**	n.d.	

a Mean values (n = 3) with standard deviations.

b Illustrative mass spectra shown in Fig. S2.

c Illustrative mass spectra shown in Fig. S3.

d Not determined. Compound present but signal-to-noise ratio for chromatographic peak < 10.

* *p* < 0.01 compared to next lower NH_4_^+^-N concentration.

** *p* < 0.01 compared to 12 g l^−1^ NH_4_^+^-N.

The strains were cultivated in different concentrations of NH_4_^+^-N and Na^+^, as indicated.

### Quantification of glycine betaine and N^ε^-acetyl-β-lysine

The concentrations of glycine betaine and N^ε^-acetyl-β-lysine were subsequently determined using deuterium labelled reference compounds, i.e. glycine betaine-*d_9_* and N^ε^-acetyl-*d_3_*-β-lysine. For all isolates, the highest concentrations of glycine-betaine were measured in cultures with 12 g l^−1^ Na^+^, ranging from 6.3 µg ml^−1^ in cultures of *M. bourgensis* MS2^T^ to the significantly lower 3.9 µg ml^−1^ (*p* < 0.02) in cultures of *M. submarinus* Nankai-1^T^ (Table 1, Fig. 4). During growth with ammonium salt at the same concentration (12 g l^−1^ NH_4_^+^-N) the levels of glycine betaine was in average ca 100 ng ml^−1^ and varied insignificantly (*p* > 0.01) between the cultures growing at different levels of NH_4_^+^-N or Na^+^. In contrast, in *M. submarinus* Nankai-1^T^ the concentration of glycine-betaine was more than 30-fold higher (2.5 µg ml^−1^, *p* < 0.01) in cultures with 12 g l^−1^ NH_4_^+^-N, compared to cultures with 0.1 g l^−1^ NH_4_^+^-N (77 ng ml^−1^). In *M. bourgensis* MS2^T^ cultures, the situation was the reversed with 10-fold higher concentration of glycine betaine in cultures with low NH_4_^+^-N levels than in high NH_4_^+^-N level cultures.

The concentration of N^ε^-acetyl-β-lysine was overall highest in *M. bourgensis* MAB2, with an increasing trend with increasing ammonium levels, reaching 6.6 µg ml^−1^ at 12 g l^−1^. For *M. submarinus* Nankai-1^T^ the level at the same ammonium concentration was ca 16-fold lower (*p* < 0.01) and significantly (*p* < 0.01) lower as compared to cultures with 12 g l^−1^ Na^+^. The N^ε^-acetyl-β-lysine concentration in *M. bourgensis* MS2^T^ was too low for quantification.

## Discussion

### Accumulation and biosynthesis of N^ε^-acetyl-β-lysine

The methanogens *Methanoculleus bourgensis* strains MAB1, MAB, MAB3, and BA1, originating from high-ammonia biogas digesters, accumulated N^ε^-acetyl-β-lysine in response to elevated level of ammonium salt. This response was not observed in the reference strains *Methanoculleus bourgensis* MS2^T^ and *Methanoculleus submarinus* Nankai-1^T^. Notably, *M. bourgensis* MAB2 exhibited a clear increase in intracellular N^ε^-acetyl-β-lysine with rising levels of ammonium chloride, representing the first documented case of explicit accumulation of this osmolyte in a methanogenic pure cultures in response to elevated levels of this salt. The compound was synthesised de novo, as LC-MS analysis of sterile medium confirmed the absence of N^ε^-acetyl-β-lysine. As increasing ammonium chloride simultaneously elevated free ammonia to inhibitory levels, the present design cannot disentangle the relative effects of ammonium salt versus free ammonia stress. Consequently, it remains unclear whether the accumulation was driven primarily by ionic (salt) stress, ammonia toxicity, or a combination of both.

Previous studies have reported N^ε^-acetyl-β-lysine accumulation in methanogens predominantly in response to sodium salt (>0.5 M NaCl), such as *Methanosarcina thermophila, Methanogenium cariaci, Methanococcus thermolithotrophicus*, and different species within genus *Methanohalophilus* (Sowers et al. 1990, Lai et al. 1991, Roesser and Müller 2001, Robertson et al. 1992b). Consistent with this, both *M. bourgensis* sp. MAB2 and *M. submarinus* Nankai-1^T^ accumulated N^ε^-acetyl-β-lysine at 12 g L⁻¹ Na⁺ (0.52 M). Accumulation at lower salt concentrations (4-40 mM NaCl or NH_4_Cl) have previously been observed only in *Methanohalophilus* strain FDF1 (Robertson et al. 1992a).

The biosynthesis of N^ε^-acetyl-β-lysine proceeds via the activity of 2,3 amino mutase (AblA), converting α-lysine to β-lysine, followed by acetylation by the lysine acetyltransferase (AblB) (Pflüger et al. 2003). Genes encoding this complete pathway have been identified in different methanogens, such as *Methanohalophilus* (Guan et al. 2019), *Methanosarcina mazei* Gö1 (Schlegel et al. 2011), and more recently in metagenome assemble genome (MAGs) of *Methanothrix* (Gagliano et al. 2022). Consistent with the observed accumulation of N^ε^-acetyl-β-lysine, AblA and AblB genes were also detected in the genomes of strain BA1 and MAB1 (Manzoor et al. 2016, Maus et al. 2016). The genomes of *Methanoculleus* MAB2 and MAB3 are unavailable and thus the presence of these genes in these species cannot be confirmed. The genomes of *M. submarinus* Nankai-1^T^ and *M. bourgensis* MS2^T^ also both contain the complete biosynthetic pathway for N^ε^-acetyl-β-lysine (Maus et al. 2015). Nevertheless, neither strain synthesised N^ε^-acetyl-β-lysine under the condition tested. Several factors may explain this discrepancy. First, both strains displayed comparably low growth rates at elevated ammonium salt concentration, which may have resulted in lower measured osmolyte levels when expressed per ml of culture. Still, as both species accumulated glycine betaine under the same conditions, it can be concluded that production of the β-amino acid derivate was comparatively low. Second, repression of N^ε^-acetyl-β-lysine synthesis by glycine betaine, as documented in *Methanosarcina* spp. (Sowers et al. 1990), could have occurred, particularly in *M. bourgensis* MS2^T^, which accumulated the highest glycine betaine levels at high Na⁺. Third, production of N^ε^-acetyl-β-lysine has been shown to require gradual osmoadaptation, and lysine 2,3-aminomutase activity appears low in cells maintained under low-salt conditions (Martin et al. 2001). Before this study *M. bourgensis* MS2^T^ had not been exposed to high salt levels, potentially explaining the lack of response. By contrast, strains MAB1, MAB2, MAB3 and BA1 were originally isolated from high-ammonia digesters and had been cultivated long-term at elevated NH_4_⁺-N concentrations, likely facilitating physiological adaptation. Whether the ability to synthesise N^ε^-acetyl-β-lysine is conserved or variable among *M. bourgensis* subspecies remains to be clarified.

### Glycine betaine accumulation and transport

All strains accumulated glycine betaine in response to NaCl, consistent with its widespread role as compatible solute in prokaryotes (Sleator et al. 2002, Salma et al. 2020,). De novo synthesis of this osmolyte is rare in archaea, and many species instead rely on high-affinity transport systems for its uptake and accumulation (Martin et al. 1999). In line with this, *M. bourgensis* MS2^T^ lacks genes for glycine betaine biosynthesis and instead encodes an Ota-like uptake system (Maus et al. 2015), which is also present in the genomes of strains BA1 and MAB1 (Manzoor et al. 2016, Maus et al. 2016). The genomic potential of *M. submarinus* Nankai-1^T^ remains unknown due to absence of a sequenced genome. The source of glycine betaine in the medium is unclear but yeast extract is the most likely contributor, which together with sodium acetate and cysteine was the only source of organic carbon in the medium.

Responses to elevated ammonium levels varied markedly between the isolates: *M. bourgensis* MAB2 accumulated relatively low levels of glycine betaine at al investigated ammonium levels, whereas *M. submarinus* Nankai-1^T^ showed the opposite trend. *M. bourgensis* MS2^T^ reduced the accumulation of glycine betaine under high NH_4_^+^-N concentrations. These variations in osmoadaptation strategies among *Methanoculleus* spp. are consistent with observations from ammonia shock loading in manure-based AD processes, where expression analyses indicated species-specific upregulation of glycine betaine transporters (Gaspari et al. 2023).

### Implications for osmoadaptation and osmolyte addition in anaerobic digestion

The identification of divergent osmolyte strategies among closely related methanogens highlight opportunities to mitigate salt and ammonia inhibition in AD-processes by supplying specific osmolytes, or introducing pre-adapted species. Most previous work has focused on sodium toxicity, where several additives have been shown to enhance methane production, for example, amino acids and tryptone in granular sludge, which promoted accumulation of N^ε^-acetyl-β-lysine after adaptation to 20 g l^−1^ Na^+^ (Sudmalis et al. 2018). In anaerobic digestion of food waste under high-salt condition (5-35 g L^−1^ NaCl), supplementation of glycine betaine and choline increased methane production (Oh et al. 2008, Liu et al. 2019). In anaerobic membrane reactors exposed to 20–40 g l⁻¹ NaCl levels, trehalose was the primary osmolyte synthesised, yet sodium stress was mitigated only when glycine betaine was supplemented over successive feedings, while additions of N^ε^-acetyl-β-lysine or potassium were ineffective (Vyrides et al. 2010). Fewer studies have explored osmo-adaptation mechanism under elevated ammonium/ammonia levels in AD processes or strategies to alleviate such inhibition. However, supplementation with glycine betaine, MgCl_2_ and KCl improved methane production under high ammonia levels in continuous anaerobic reactors, with lasting effects attributed to enrichment of *Methanoculleus* spp. (Yan et al. 2022). Bioaugmentation with ammonia adapted *M. bourgensis* MS2^T^ have also been shown in several studies to alleviate ammonia inhibition in AD-processes (reviewed in Li et al. (2023)). To date no studies have investigated the supplementation with N^ε^-acetyl-β-lysine as a mitigation strategy for salt stress during AD. Nevertheless, upregulation of the AblB gene of two different *Methanoculleus sp*. were demonstrated during AD of cattle manure at elevated ammonia levels (5 g l^−1^ NH_4_^+^-N) (Gaspari et al. 2023). Furthermore, in syntrophic propionate-degrading enrichment cultures ’*Candidatus* Methanoculleus ammoniitolerans’ expressed the AblB gene as well as the gene trehalose 6-phosphate synthase, indicating active trehalose synthesis during cultivation under high-ammonia conditions (Weng et al. 2024). Given its association with ammonia tolerance in the present work, targeted addition of this osmolyte, or bioaugmentation with N^ε^-acetyl-β-lysine -producing methanogens, warrants further investigation.

## Conclusion

In conclusion, this study demonstrates that *M. bourgenis* MAB1, MAB2, MAB3, and BA1, isolated from high ammonia biogas processes synthesised and accumulated N^ε^-acetyl-β-lysine in response to elevated levels of ammonium- or sodium chloride (12 g l^−1^). In contrast, the type strain, *M. bourgenis* MS^T^ and the salt tolerant *M. submarinus* accumulated mainly glycine betaine under the same conditions, with only smaller levels of N^ε^-acetyl-β-lysine detected in the latter. The ability to produce N^ε^-acetyl-β-lysine therefore provides a plausible explanation for the comparably high ammonia tolerance observed for the strains isolated from the high ammonia environments. Interestingly, both *M. bourgenis* MS^T^ and *M. submarinus* harbour the AlbA and AlbB genes encoding the complete synthesis pathway for N^ε^-acetyl-β-lysine yet did not produce the compound under the conditions tested. Their inability to synthetize N^ε^-acetyl-β-lysine may reflect requirement for long term adaptation to high ammonia levels, although this hypothesis warrants further investigations. Thus, additional studies are needed to elucidate the regulatory factors underlying Nε-acetyl-β-lysine biosynthesis and to assess the extent of intraspecies variation among M. bourgensis subspecies.

## Supplementary Material

fnaf143_Supplemental_File

## References

[bib1] Bremer E, Krämer R. Responses of microorganisms to osmotic stress. Annu Rev Microbiol. 2019;73:313–34. 10.1146/annurev-micro-020518-115504. 31180805

[bib2] Capson-Tojo G, Moscoviz R, Astals S et al. Unraveling the literature chaos around free ammonia inhibition in anaerobic digestion. Renewable Sustainable Energy Rev. 2020;117:109487. 10.1016/j.rser.2019.109487.

[bib3] Duan H, He P, Zhang H et al. Metabolic regulation of mesophilic *Methanosarcina barkeri* to ammonium inhibition. Environ Sci Technol. 2022;56:8897–907. 10.1021/acs.est.2c01212. 35588324

[bib4] Feng G, Zeng Y, Wang HZ et al. *Proteiniphilum* and *Methanothrix harundinacea* became dominant acetate utilizers in a methanogenic reactor operated under strong ammonia stress. Front Microbiol. 2022;13:1098814. 10.3389/fmicb.2022.1098814. 36687577 PMC9853277

[bib5] Fotidis IA, Treu L, Angelidaki I. Enriched ammonia-tolerant methanogenic cultures as bioaugmentation inocula in continuous biomethanation processes. J Cleaner Prod. 2017;166:1305–13. 10.1016/j.jclepro.2017.08.151.

[bib6] Gagliano MC, Sampara P, Plugge CM et al. Functional insights of salinity stress-related pathways in metagenome-resolved *Methanothrix* genomes. Appl Environ Microb. 2022;88:e02449–21. 10.1128/aem.02449-21. PMC9128505 35477253

[bib50_112_301725] Gaspari M, Ghiotto G, Centurion VB et al. Decoding microbial responses to ammonia shock loads in biogas reactors through metagenomics and metatranscriptomics. Environ Sci Technol. 2023; 58:591–602. 10.1021/acs.est.3c07840. 38112274 PMC10785759

[bib7] Guan Y, Ngugi DK, Vinu M et al. Comparative genomics of the genus *Methanohalophilus*, including a newly isolated strain from Kebrit deep in the Red sea. Front Microbiol. 2019;10. 10.3389/fmicb.2019.00839. PMC6491703 31068917

[bib8] Halim MFA, Day LA, Costa KC. Formate-dependent heterodisulfide reduction in a Methanomicrobiales Archaeon. Appl Environ Microb. 2021;87:1–14. 10.1128/aem.02698-20. PMC8104993 33361366

[bib9] Houwen FP, Dijkema C, Schoenmakers CHH et al. 13C-NMR study of propionate degradation by a methanogenic coculture. FEMS Microbiol Lett. 1987;41:269–74. 10.1111/j.1574-6968.1987.tb02209.x.

[bib10] Kato S, Sasaki K, Watanabe K et al. Physiological and transriptomic analyses of the thermophilic, aceticlastic methanogen *Methanosaeta thermophila* responding to ammonia stress. Microb Environ. 2014;29:162–7. 10.1264/jsme2.me14021. PMC4103522 24920170

[bib11] Kougias PG, Angelidaki I. Biogas and its opportunities-A review. Front Environ Sci Eng. 2018;12:14. 10.1007/s11783-018-1037-8.

[bib12] Lai MC, Sowers KR, Robertson DE et al. Distribution of compatible solutes in the halophilic methanogenic archaebacteria. J Bacteriol. 1991;173:5352–8. 10.1128/jb.173.17.5352-5358.1991. 1909318 PMC208245

[bib13] Lee J, Kim E, Hwang S. Effects of inhibitions by sodium ion and ammonia and different inocula on acetate-utilizing methanogenesis: methanogenic activity and succession of methanogens. Bioresour Technol. 2021;334:125202. 10.1016/j.biortech.2021.125202. 33957457

[bib14] Li Z-Y, Inoue D, Ike M. Mitigating ammonia-inhibition in anaerobic digestion by bioaugmentation: a review. J Water Process Eng. 2023;52:103506. 10.1016/j.jwpe.2023.103506.

[bib15] Liu Y, Yuan Y, Wang W et al. Effects of adding osmoprotectant on anaerobic digestion of kitchen waste with high level of salinity. J Biosci Bioeng. 2019;128:723–32. 10.1016/j.jbiosc.2019.05.011. 31466824

[bib16] Manzoor S, Schnürer A, Bongcam-Rudloff E et al. Complete genome sequence of *Methanoculleus bourgensis* strain MAB1, the syntrophic partner of mesophilic acetate-oxidising bacteria (SAOB). Stand in Genomic Sci. 2016;11:80. 10.1186/s40793-016-0199-x. 27777650 PMC5062929

[bib17] Martin DD, Ciulla RA, Roberts MF. Osmoadaptation in archaea. Appl Environ Microb. 1999;65:1815–25. 10.1128/AEM.65.5.1815-1825.1999. PMC91261 10223964

[bib51_998_310625] Martin DD, Ciulla RA, Robinson PM et al. Switching osmolyte strategies: response of Methanococcus thermolithotrophicus to changes in external NaCl. Biochim Biophys Acta. 2001;1524:1–10. 11078952 10.1016/s0304-4165(00)00131-8

[bib49_504_300825] Martin DD, Ciulla RA, Robinson PM. Switching osmolyte strategies: response of Methanococcus thermolithotrophicus to changes in external NaCl. Biochim Biophys Acta. 2001;1524:1–10. 11078952 10.1016/s0304-4165(00)00131-8

[bib18] Maus I, Wibberg D, Stantscheff R et al. Insights into the annotated genome sequence of *Methanoculleus bourgensis* MS2^T^, related to dominant methanogens in biogas-producing plants. J Biotechnol. 2015;201:43–53. 10.1016/j.jbiotec.2014.11.020. 25455016

[bib19] Maus I, Wibberg D, Winkler A et al. Complete genome sequence of the methanogen *Methanoculleus bourgensis* BA1 isolated from a biogas reactor. Genome Announc. 2016;4:e00568–16. 10.1128/genomeA.00568-16. 27340059 PMC4919398

[bib20] Meiboom S, Gill D. Modified spin-echo method for measuring nuclear relaxation times. Rev Sci Instr. 1958;29:688–91. 10.1063/1.1716296.

[bib21] Merrick MJ, Edwards RA. Nitrogen control in bacteria. Microbiol Rev. 1995;59:604–22. 10.1128/mr.59.4.604-622.1995. 8531888 PMC239390

[bib22] Mikucki JA, Liu YT, Delwiche M et al. Isolation of a methanogen from deep marine sediments that contain methane hydrates, and description of *Methanoculleus submarinus* sp nov. Appl Environ Microb. 2003;69:3311–6. 10.1128/aem.69.6.3311-3316.2003. PMC161549 12788731

[bib23] Oh G, Zhang L, Jahng D. Osmoprotectants enhance methane production from the anaerobic digestion of food waste containing a high content of salt. J of Chemical Tech & Biotech. 2008;83:1204–10. 10.1002/jctb.1923.

[bib24] Ollivier BM, Mah RA, Garcia JL et al. Isolation and characterization of *Methanogenium bourgense* sp. nov. Int J Syst Bacteriol. 1986;36:297–301. 10.1099/00207713-36-2-297.

[bib25] Pflüger K, Baumann S, Gottschalk G et al. Lysine-2,3-aminomutase and β-lysine acetyltransferase genes of methanogenic archaea are salt induced and are essential for the biosynthesis of Nε-acetyl-β-lysine and growth at high salinity. Appl Environ Microb. 2003;69:6047–55. 10.1128/aem.69.10.6047-6055.2003. PMC201229 14532061

[bib26] Robertson DE, Lai MC, Gunsalus RP et al. Composition, variation, and dynamics of major osmotic solutes in *Methanohalophilus* strain FDF1. Appl Environ Microb. 1992;58:2438–43. 10.1128/aem.58.8.2438-2443.1992. PMC195800 16348748

[bib27] Robertson DE, Noll D, Roberts MF. Free amino acid dynamics in marine methanogens. Beta-amino acids as compatible solutes. J Biol Chem. 1992;267:14893–901. 10.1016/S0021-9258(18)42124-2. 1353078

[bib28] Roesser M, Müller V. Osmoadaptation in bacteria and archaea: common principles and differences. Environ Microbiol. 2001;3:743–54. 10.1046/j.1462-2920.2001.00252.x. 11846768

[bib29] Salma M, Abdulla MK, Samina M. Osmoadaptation in halophilic bacteria and archaea. Resear J Biotechnol. 2020;15:154–61.

[bib30] Schlegel K, Müller V. Osmoadaptation in methanogenic Archaea: physiology, genetics, and regulation in *Methanosarcina mazei* Gö1. In: Horikoshi K (ed.) Extremophiles Handbook, Tokyo: Springer Japan, 2011,327–42. 10.1007/978-4-431-53898-1_15.

[bib31] Schnürer A, Nordberg A. Ammonia, a selective agent for methane production by syntrophic acetate oxidation at mesophilic temperature. Water Sci Technol. 2008;57:735–40. 10.2166/wst.2008.097. 18401146

[bib32] Schnürer A, Zellner G, Svensson BH. Mesophilic syntrophic acetate oxidation during methane formation in biogas reactors. FEMS Microbiol Ecol. 1999;29:249–61. 10.1016/S0168-6496(99)00016-1.

[bib33] Schnürer A. Biogas production: microbiology and technology *Advances in Biochemical Engineering/Biotechnology*. 2016;156:195–234. 10.1007/10_2016_5 27432246

[bib34] Sleator RD, Hill C. Bacterial osmoadaptation: the role of osmolytes in bacterial stress and virulence. FEMS Microbiol Rev. 2002;26:49–71. 10.1111/j.1574-6976.2002.tb00598.x. 12007642

[bib35] Sowers KR, Robertson DE, Noll D et al. N^ε^-Acetyl-β-lysine: an osmolyte synthesized by methanogenic archaebacteria. Proc Natl Acad Sci USA. 1990;87:9083–7. 10.1073/pnas.87.23.9083. 2123548 PMC55108

[bib36] Sprott GD, Patel GB. Ammonia toxicity in pure cultures of methanogenic bacteria. Syst Appl Microbiol. 1986;7:358–63. 10.1016/S0723-2020(86)80034-0.

[bib37] Sudmalis D, Millah SK, Gagliano MC et al. The potential of osmolytes and their precursors to alleviate osmotic stress of anaerobic granular sludge. Water Res. 2018;147:142–51. 10.1016/j.watres.2018.09.059. 30308373

[bib38] Tian HL, Mancini E, Treu L et al. Bioaugmentation strategy for overcoming ammonia inhibition during biomethanation of a protein-rich substrate. Chemosphere. 2019;231:415–22. 10.1016/j.chemosphere.2019.05.140. 31146133

[bib39] Triadó-Margarit X, Vila X, Galinski EA. Osmoadaptative accumulation of Nɛ-acetyl-β-lysine in green sulfur bacteria and *Bacillus cereus* CECT 148T. FEMS Microbiol Lett. 2011;318:159–67. 10.1111/j.1574-6968.2011.02254.x. 21371089

[bib40] Vyrides I, Santos H, Mingote A et al. Are compatible solutes compatible with biological treatment of saline wastewater? batch and continuous studies using submerged anaerobic membrane bioreactors (SAMBRs). Environ Sci Technol. 2010;44:7437–42. 10.1021/es903981k. 20831155

[bib41] Wang H, Fotidis IA, Angelidaki I. Ammonia effect on hydrogenotrophic methanogens and syntrophic acetate oxidizing bacteria. FEMS Microbiol Ecol. 2015;91:1–8. 10.1093/femsec/fiv130. 26490748

[bib42] Wang Z, Wang S, Hu Y et al. Distinguishing responses of acetoclastic and hydrogenotrophic methanogens to ammonia stress in mesophilic mixed cultures. Water Res. 2022;224:119029. 10.1016/j.watres.2022.119029. 36099760

[bib43] Weinisch L, Kühner S, Roth R et al. Identification of osmoadaptive strategies in the halophile, heterotrophic ciliate *Schmidingerothrix salinarum*. PLOS Biol. 2018;16:e2003892. 10.1371/journal.pbio.2003892. 29357351 PMC5794333

[bib44] Welsh DT. Ecological significance of compatible solute accumulation by micro-organisms: from single cells to global climate. FEMS Microbiol Rev. 2000;24:263–90. 10.1111/j.1574-6976.2000.tb00542.x. 10841973

[bib45] Weng N, Singh A, Ohlsson JA et al. Catabolism and interactions of syntrophic propionate- and acetate oxidizing microorganisms under mesophilic, high-ammonia conditions. Front Microbiol. 2024;15. 10.3389/fmicb.2024.1389257. PMC11201294 38933034

[bib46] Westerholm M, Moestedt J, Schnürer A. Biogas production through syntrophic acetate oxidation and deliberate operating strategies for improved digester performance. Appl Energy. 2016;179:124–35. 10.1016/j.apenergy.2016.06.061.

[bib47] Westerholm M, Roos S, Schnürer A. *Syntrophaceticus schinkii* gen. nov., sp. nov., an anaerobic, syntrophic acetate-oxidizing bacterium isolated from a mesophilic anaerobic filter. FEMS Microbiol Lett. 2010;309:no–no. 10.1111/j.1574-6968.2010.02023.x. 20546311

[bib48] Yan YX, Yan M, Angelidaki I et al. Osmoprotectants boost adaptation and protect methanogenic microbiome during ammonia toxicity events in continuous processes. Bioresour Technol. 2022;364:128106. 10.1016/j.biortech.2022.128106. 36243262

