# Supplementary material for: Nε-acetyl-β-lysine or glycine betaine as compatible solutes in response to increasing ammonia in Methanoculleus sp strains
Source: FEMS Microbiol Lett. 2025 Dec 24;373:fnaf143. doi: 10.1093/femsle/fnaf143 (PMC12776343; doi:10.1093/femsle/fnaf143)
Supplement: fnaf143_Supplemental_File [file fnaf143_supplemental_file.docx]

**Supplementary Information:**

**N^ε^-acetyl-β-lysine or glycine betaine as compatible solutes in response to increasing ammonia in *Methanoculleus sp* strains**

Anna Schnürer^1*^, Maria Westerholm and Anders Broberg

*Department of Molecular Sciences, Swedish University of Agricultural Sciences, Uppsala BioCenter, Box 7025, SE-750 07 Uppsala, Sweden,*

Keywords; methanogen, ammonia stress, compatible solute, glycine betaine, N^ε^-acetyl-β-lysine, HR-MAS NMR

**Corresponding author**:

Anna Schnürer

Department of Molecular Sciences, BioCenter

Swedish University of Agricultural Sciences, SLU

Box 7015, 750 07 Uppsala, Sweden

Email: [anna.schnurer@slu.se](mailto:anna.schnurer@slu.se)

Tel: +46734231517


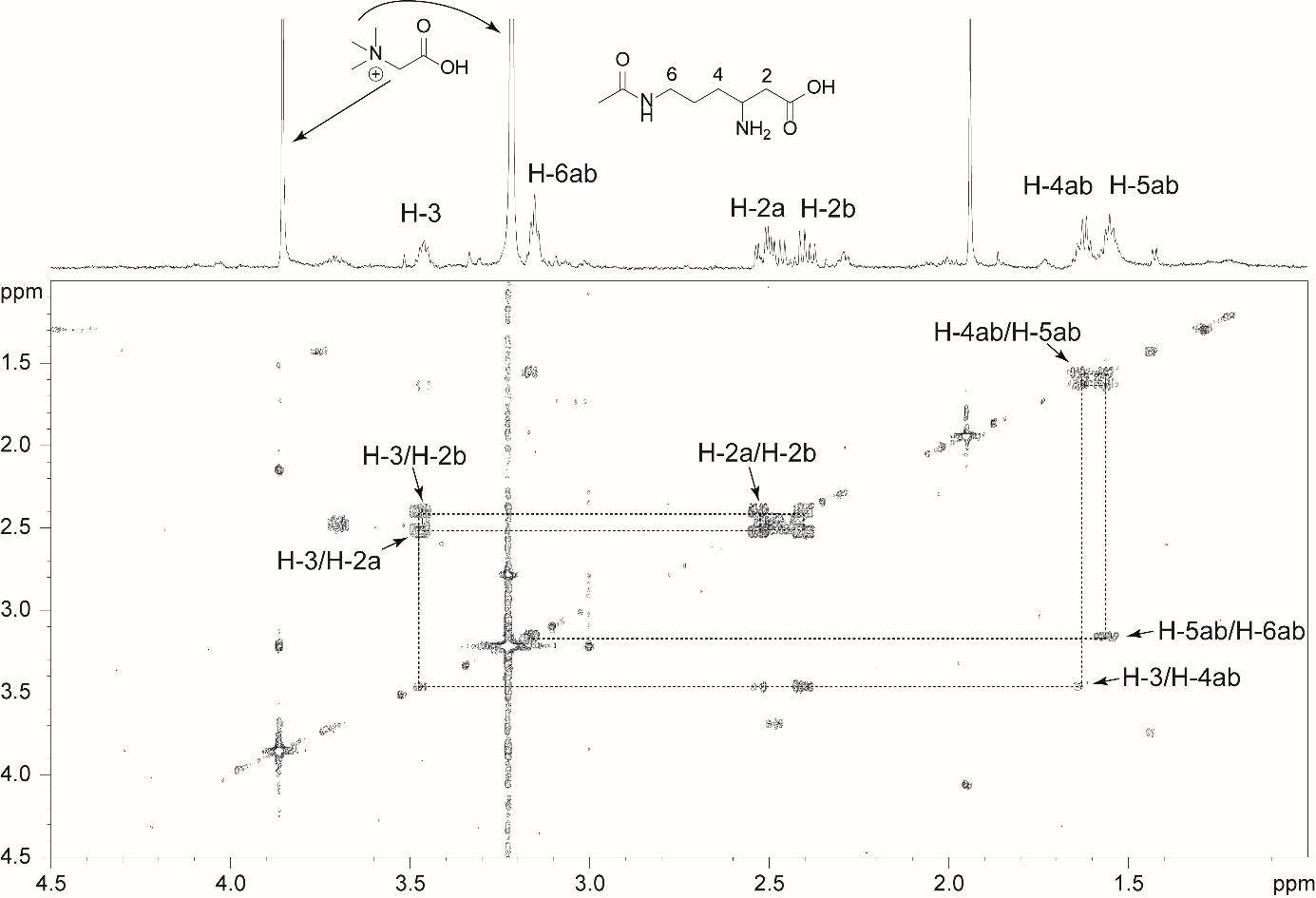


**Fig. S1.** HR-MAS NMR COSY spectrum of intact cells of *Methanoculleus bourgensis* BA1, illustrating how the spin-system of N^ε^-acetyl-β-lysine could be established by the observed cross-peaks.

**Synthesis of deuterium labelled internal standards***Glycine betaine-d_9_*: Glycine (358 mg, 4.8 mmol), CH_3_OH (50 ml), MeI-*d*_3_ (4.6 g, 32 mmol) and Dowex 1 (HCO_3_^-^ form) was mixed in a round bottomed flask, and the mixture was agitated for 17 h at room temperature. The suspension was filtered (glass filter) and dried under reduced pressure, yielding 114 mg of a 1:1 mixture of glycine and glycine betaine-*d*_9_, as judged by ^1^H-NMR and LC-MS. The crude product was dissolved in H_2_O (1 ml) and the solution was passed through an anion exchange column (Dowex 1, OH^-^) column. The column was washed with 2 + 2 ml H_2_O, and the combined fractions were lyophilized, yielding 53 mg of crude Glycine betaine-d_9_. Finally, the compound was purified by preparative HPLC on a HyperCarb column [100 × 21.2 mm Hypercarb column (5 µm; ThermoQuest Runcorn, Cheshire, United Kingdom] using H_2_O as solvent, at 10 ml/min, and with UV-detection at 210 nm. Fractions containing the compound, as indicated by LC-MS, were pooled and lyophilized. Compound: 47.6 mg; ^1^H-NMR (D_2_O, 400 MHz) δ 3.88 (2H, s, H-2ab); ^13^C-NMR (D_2_O, 100 MHz) δ 170.0 (CO, C-1), δ 66.8 (CH_2_, C-2), δ 53.2 (CD_3_, N-*C*D_3_); HRFABMS *m/z* 127.1426 M^+^ (calcd for C_5_H_3_D_9_NO_2_ 127**.**1427).

*N^ε^-acetyl-d_3_-L-β-lysine: N*,*N*-Dicyclohexylammonium (*3S*)*-*6-(benzyloxycarbonylamino)-3-(tert-butoxycarbonylamino) hexanoate (507 mg, 0.902 mmol) was partitioned between EtOAc and aqueous 0.1 M HCl, and the organic phase washed twice with H_2_O. Following evaporation of the solvent, the residue was dissolved in 25 ml CH_3_OH in a round-bottomed flask, and 10% PdI (110 mg) was added. After flushing with N_2_ (g), the flask was filled with H_2_ (g) at ambient pressure and temperature. The hydrogenolysis was allowed to run with constant agitation for 2 h. The resulting mixture was filtered (first 00H filter; Munktell, Sweden; and then 0.45 μm syringe filter; Sarstedt, Germany), and dried under reduced pressure, yielding 260 mg (1.1 mmol) of (*3S*)-6-amino-3-(tert-butoxycarbonylamino) hexanoic acid. This compound (260 mg, 1.1 mmol) was treated with 4 equivalents of AcCl-*d*_3_ in pyridine (10 ml) at 0°C for 30 min, in the presence of molecular sieve (4 Å), followed by 30 min at room temperature. The reaction was quenched by the addition of CH_3_OH (5 ml) diluted with EtOAc. The organic phase was washed twice with aqueous 0.1 M HCl and twice with H_2_O, dried over Na_2_SO_4_, and dried under reduced pressure. The reaction product was partitioned between dichloromethane and 0.1% ammonia in H_2_O. The H_2_O phase was acidified by addition of aqueous 1 M HCl, and evaporation under reduced pressured yielded 190 mg crude N^ε^-(acetyl-d_3_)-L-β-lysine. This crude product was analyzed by NMR and LC-MS, verifying the dominance of the compound but also significant amounts of ammonium chloride and pyridinium chloride. A portion of the crude compound was purified by preparative HPLC on a HyperCarb porous graphitic carbon [100 × 21.2 mm Hypercarb column (5 µm; ThermoQuest Runcorn, Cheshire, United Kingdom] using aqueous 20% CH_3_OH as solvent, at 10 ml/min, and with UV-detection at 210 nm. Fractions containing N^ε^-(acetyl-d_3_)-L-β-lysine, as indicated by LC-MS, were pooled and lyophilized. Compound: 16.2 mg; white powder; [α]^20^_D_ +20.1 (*c* 0.364, H_2_O); ^1^H-NMR (D_2_O, 400 MHz) δ 3.54 (1H, m, H-3), δ 3.20 (2H, t, *J*=6.6 Hz, H-6ab), δ 2.63 (1H, dd, J=16.8 Hz, J=4.7 Hz, H-2a), δ 2.50 (1H, dd, J=16.8 Hz, J= 8.2 Hz, H-2b), δ 1.68 (2H, m, H-4ab), δ 1.60 (2H, m, H-5ab); ^13^C-NMR (D_2_O, 100 MHz) δ 177.9 (CO, C-1), δ 175.0 (CO, *C*OCD_3_), δ 49.7 (CH, C-3), δ 39.6 (CH_2_, C-6), δ 38.6 (CH_2_, C-2), δ 30.2 (CH_2_, C-4), δ 25.2 (CH_2_, C-5), δ 22.0 (CD_3_, CO*C*D_3_); HRFABMS *m/z* 192.1427 (M+H)^+^ (calcd for C_8_H_14_D_3_N_2_O_3_ 192.1422).


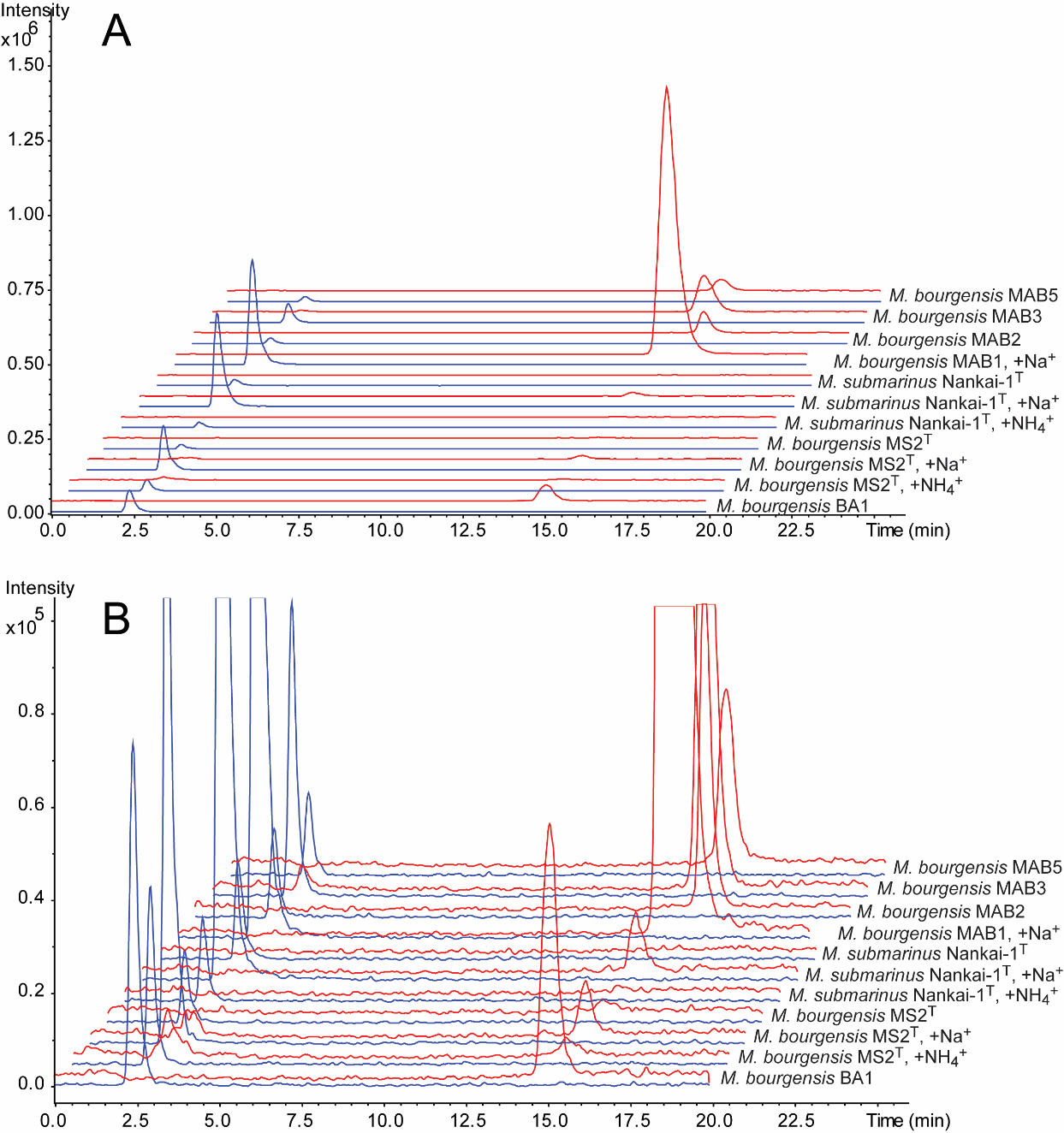


**Fig. S2.** Extracted ion chromatograms from LC-MS analysis of cultures of *Methanoculleus* sp. strains showing M^+^ for glycine betaine (blue chromatograms, *m/z* 118.1, t_R_ 2.2 min) and M+H^+^ for N^ε^-acetyl-β-lysine (red chromatograms, *m/z* 189.2, t_R_ 14.9 min). These analyses were made without internal standards. Panel A shows the full intensity window, whereas panel B shows the same chromatograms but zoomed in along the intensity axis. Note, these analyses were run at different conditions compared to the subsequent analyses using internal standards for quantification (see Materials and Methods for details). Strain MAB5, MAB2, MAB1, BA1 were grown at 12 g l^-1^ NH_4_^+^-N and MAB2 at 12 g Na^+^. *M. submarinus* and M. bourgensis MST^2^ were grown with and without salts.
